# Supplementary material for: IS21 family transposase cleaved donor complex traps two right-handed superhelical crossings
Source: Nat Commun. 2023 Apr 22;14:2335. doi: 10.1038/s41467-023-38071-x (PMC10122671; doi:10.1038/s41467-023-38071-x)
Supplement: Supplementary file 1 — Supplementary information [file 41467_2023_38071_MOESM1_ESM.pdf]

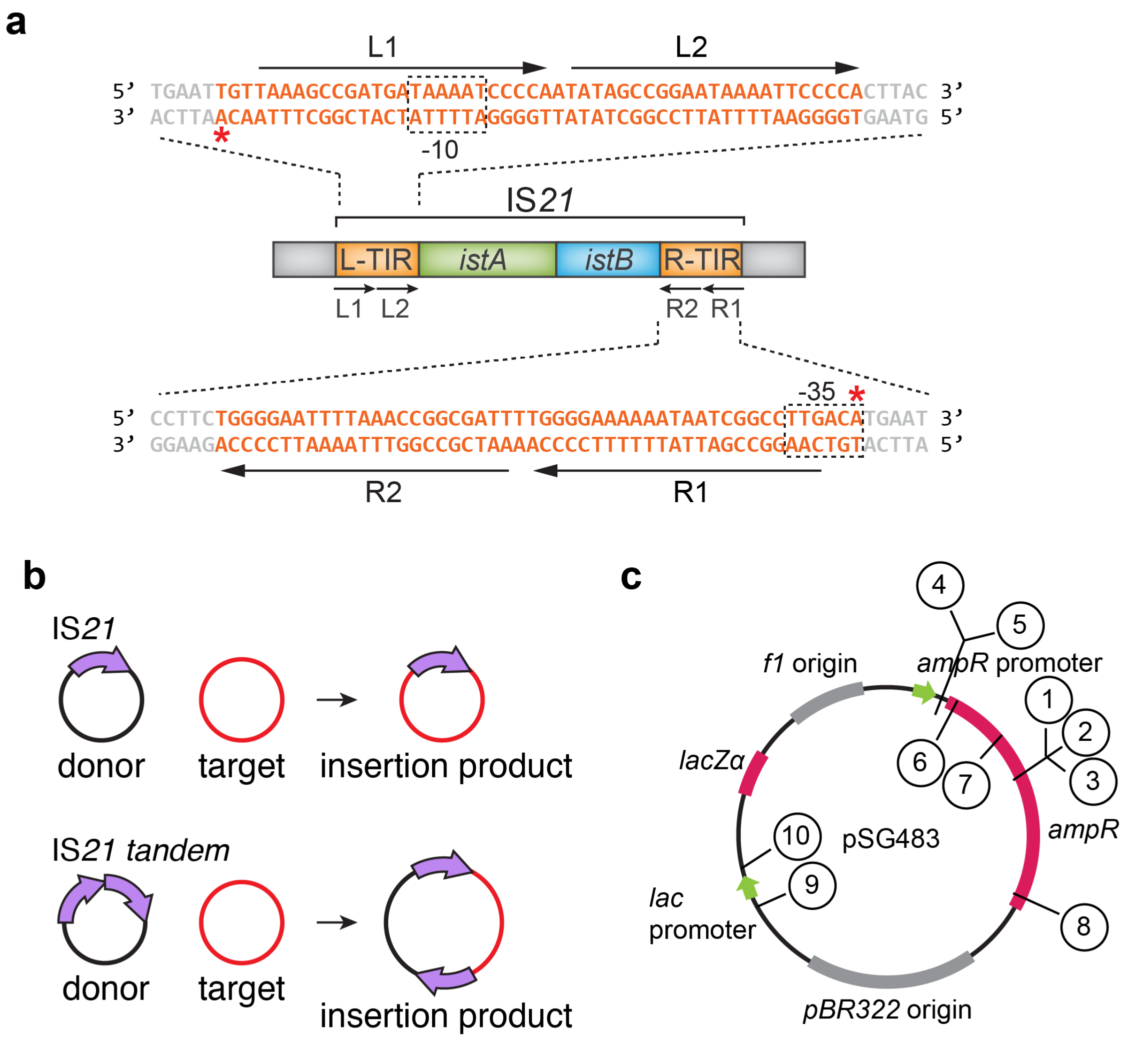

Supplementary Fig. 1 | Organization and detailed sequence of IS*21* transposon ends and plasmid insertion sites. a, Sequence of the transposon ends of the IS*21* family member IS*5376*. Each of the terminal inverted repeats contains two 23 bp-long direct repeats (L1 and L2, and R1 and R2 respectively). The TIRs also contain -10 and -35 promoter sequences that are highlighted with black dotted rectangles. The cleavage site (3' adenine) is marked with a red asterisk. **b,** Schematic of two of the main substrates used by the IstA transposase. **c,** Location of ten insertions in the pSG483 plasmid used as target DNA for *in vitro* integration assays.

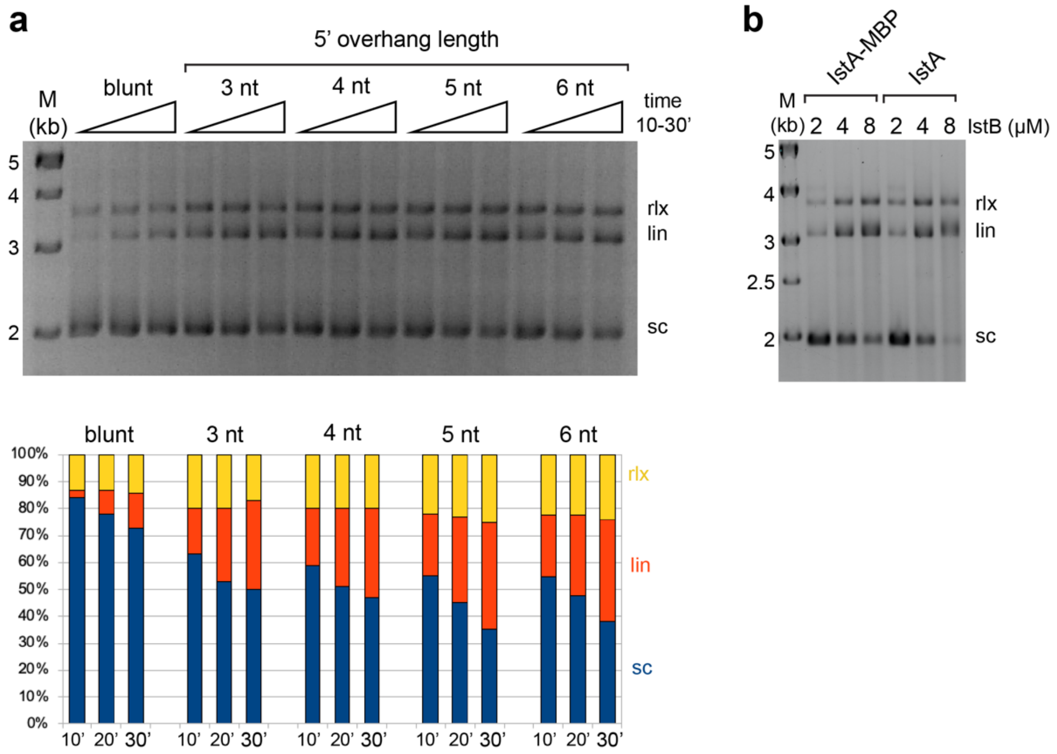

Supplementary Fig. 2 | Effects of the length of the donor 5' overhang and the presence of MBP on the activity of IstA. a, *in vitro* integration reactions in the presence of different lengths of the 5' overhang (upper panel), and quantification of the bands in the native gel (lower panel). Product of the reaction labeled as supercoiled (sc, blue), linear (lin, red) and relaxed (rlx, yellow) DNA. Molecular weight marker (in kb) indicated as M. This experiment was repeated three independent times. Source data are provided as a Source Data file. **b,** Integration reactions with the IstA-MBP fusion protein used in fluorescence anisotropy experiments show that the activity of the tagged enzyme is comparable to that of wildtype IstA. Molecular weight marker (in kb) indicated as M. This experiment was repeated three independent times. Source data are provided as a Source Data file.

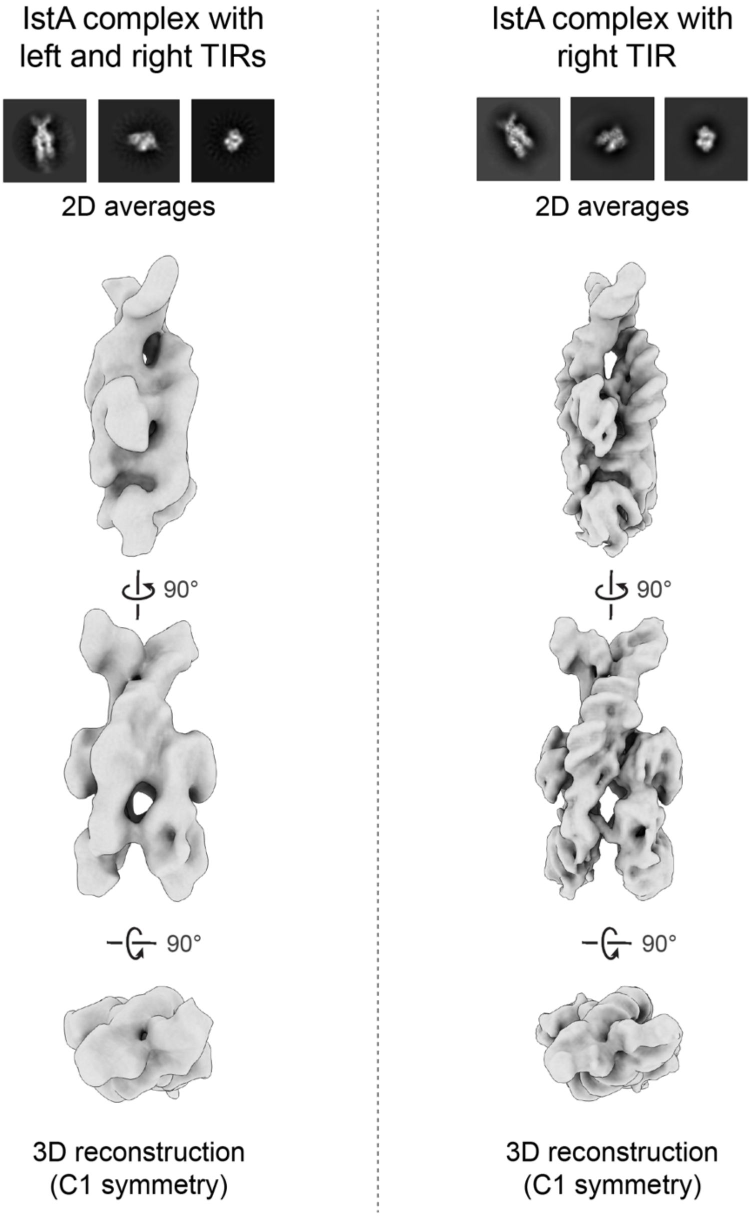

Supplementary Fig. 3 | Cryo-EM structures of IstA in complex with the isolated right TIR and a stoichiometric mixture of left and right TIRs. The 2D class averages and different orientations of the 3D reconstructions obtaining without imposing symmetry (C1) indicate that the transposase adopts a similar configuration when bound to the isolated right TIR or a equimolar mix of left and right TIRs.

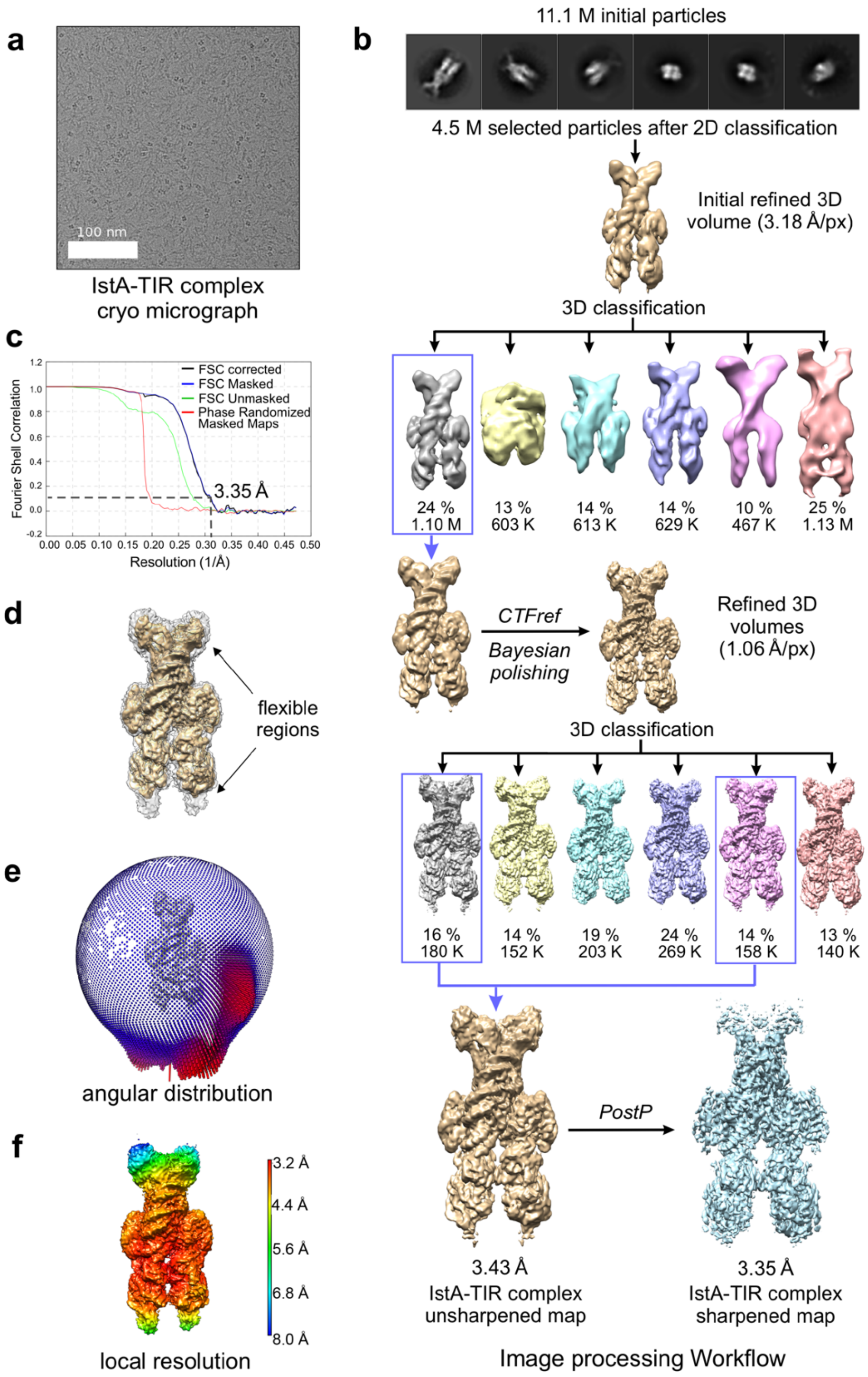

Supplementary Fig. 4 | High-resolution cryo-EM analysis and image processing. **a,** Representative cryo-EM micrograph of IstA•TIR complexes (from a total of 7,215 images). **b,** Image processing workflow. **c,** Fourier Shell Correlation of the final density map. Source data are provided as a Source Data file. **d,** Unsharpened map showed at two different thresholds (0.0035 and 0.01). The upper and lower regions of the density are particularly flexible. **e,** Angular distribution plot showing the range of observed particle orientations. **f,** Local resolution of the unsharpened map (same as panel (d); 0.0035 threshold).

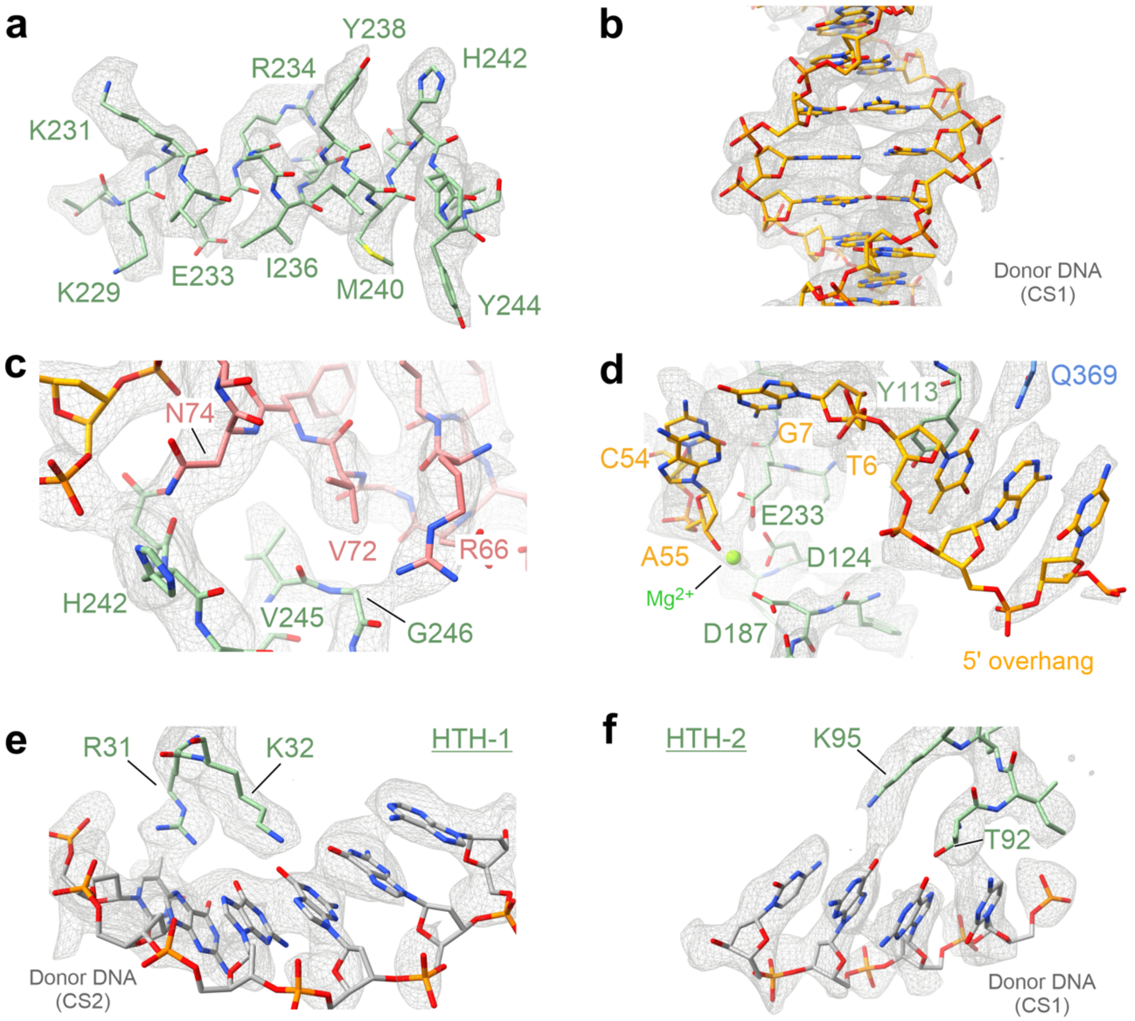

Supplementary Fig. 5 | Detailed views relevant regions of the map and atomic model. a, Protein region comprising residues 228-245 in the DDE domain of the catalytic subunits (density map is contoured at a threshold value of 0.025 in all panels). **b,** CS1 DNA region (DA10-DT51 and complementary DG16-DC45 nucleotides). **c,** Interface between upper and lower chains showing protein-protein interaction details. **d,** IstA active site. Catalytic triad and some key elements implicated in stabilizing the flipped 5' overhang are labelled. **e,** Specific recognition of the conserved DNA sequence 2 (CS2) by residues R31 and K32 located at the HTH-1 domain. **f,** Interactions between T92 and K95 (HTH-2 domain) and the CS1 of donor DNA.

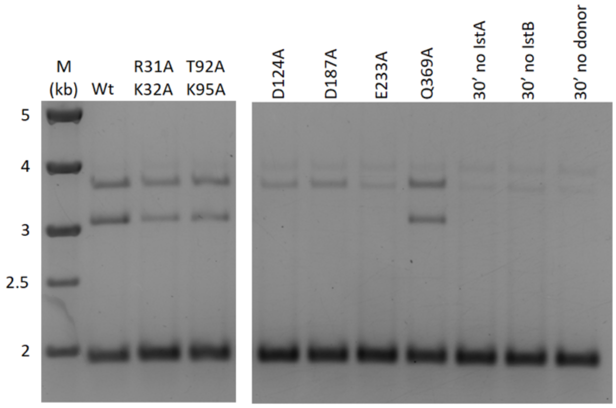

Supplementary Fig. 6 | Effect of IstA mutants on the integration reaction. Representative native agarose gel of the effect of the catalytic and DNA-binding mutants used in this study. This experiment was repeated four times (except for E233A that was repeated three times). Source data are provided as a Source Data file.

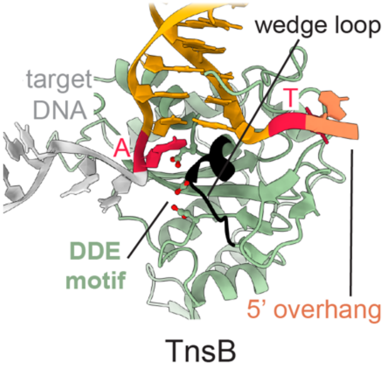

Supplementary Fig. 7 | Comparison between the active sites of the PFV and SIVrcm integrases and TnsB. The PFV and SIVrcm integrases and the Tn7 transposase (PDB IDs 3OY9 [https://doi.org/10.2210/pdb3OY9/pdb], 6RWL [https://doi.org/10.2210/pdb6RWL/pdb] and 8AA5 [https://doi.org/10.2210/pdb8AA5/pdb], respectively) use an equivalent wedge loop to flip the thymine complementary to the reactive 3’ adenosine and the 5’ overhang.

a

<u>IS5376 transposon ends</u>:

```
L1:   TAAAGCCGATGATAAAATCCCCA (4-26)
L2:   TATAGCCGGAATAAAATTCCCCA (28-52)
R1:   CAAGGCCGATTATTTTTTCCCCA (2082-2104) *
R2:   AATCGCCGGTTTAAAATTCCCCA (2058-2080) *
          CS1              CS2
```

* complementary strand

b

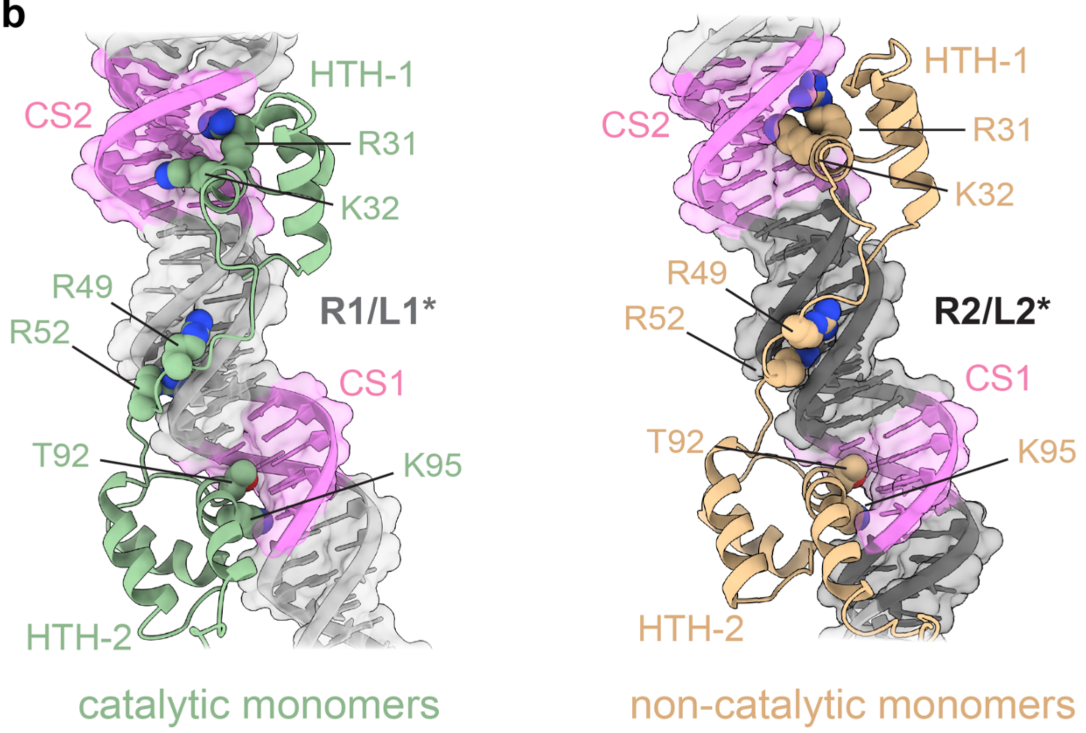

Supplementary Fig. 8 | Recognition of the multiple terminal repeats by IstA.
a, Alignment of the four repeats (L1, L2, R1 and R2) reveals the presence of two conserved sequence motifs (CS1 and CS2). These regions are contacted by the HTH-1 and HTH-2 domains. **b,** The HTH domains of the catalytic and non-catalytic IstA monomers interact with the multiple terminal repeats (R1 and R2, respectively) using similar protein-DNA contacts. The left transposon end was not used in the high-resolution structure but, due to the elevated sequence identity that exists between the left and right transposon ends, the interaction with the L1 and L2 repeats (marked with asterisk) is likely established using similar contacts.

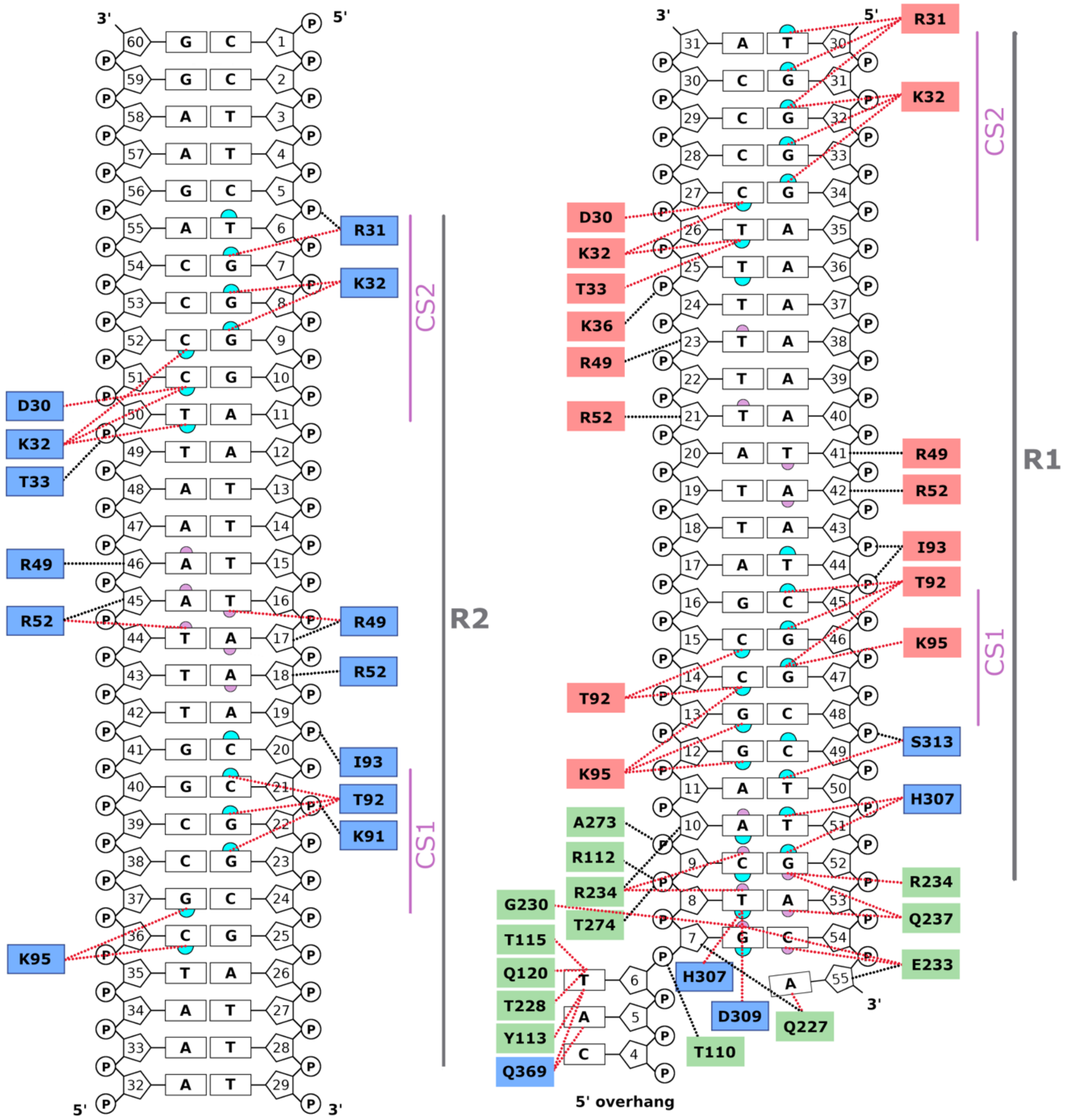

Supplementary Fig. 9 | Diagram of protein–DNA interactions. Schematic of interactions of IstA with a transposon end (duplex comprising chains E and F). The DNA is divided into two parts for clarity. Unspecific contacts with the sugar and phosphate groups are indicated with black dashed lines. Specific side chain-base interactions are shown with red dashed lines (blue and pink semicircles represent major and minor groove contacts respectively). Amino acids are colored based on chain identity (color code as per Fig. 3a).

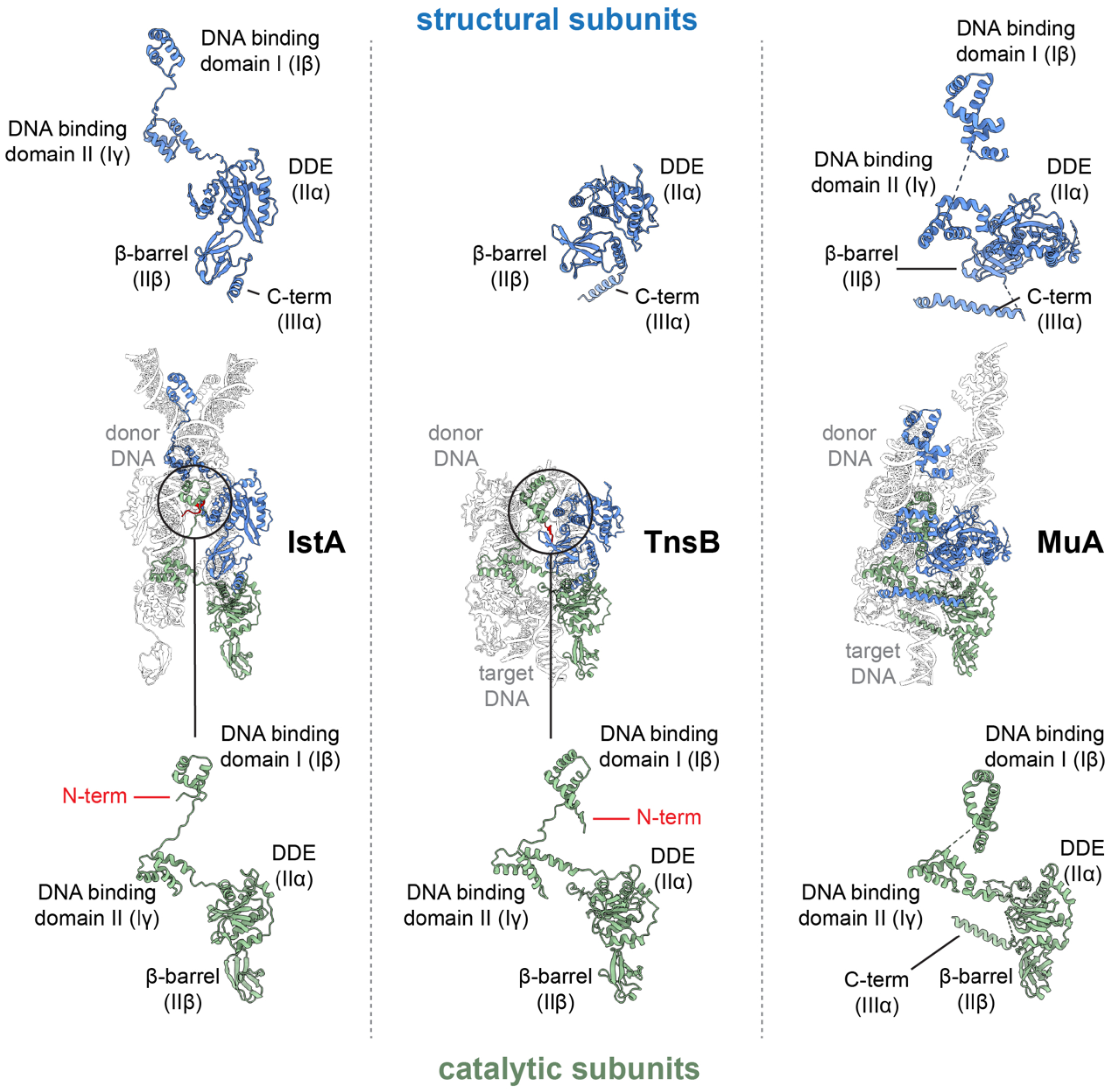

Supplementary Fig. 10 | Domain organization of the IstA, TnsB and MuA structures.

Structures of IstA, TnsB (PDB ID 8AA5 [https://doi.org/10.2210/pdb8AA5/pdb]) and MuA (PDB ID 4FCY [https://doi.org/10.2210/pdb4FCY/pdb]). Only one structural (blue) and catalytic (green) monomers have been highlighted in each structure for clarity. The three structures have been aligned using the catalytic subunit as reference. In the case of IstA and TnsB, the N-terminal region of the first DNA binding domain establishes interactions with other subunits at the core of the tetrameric complex (MuA adopts a significantly more extended configuration).

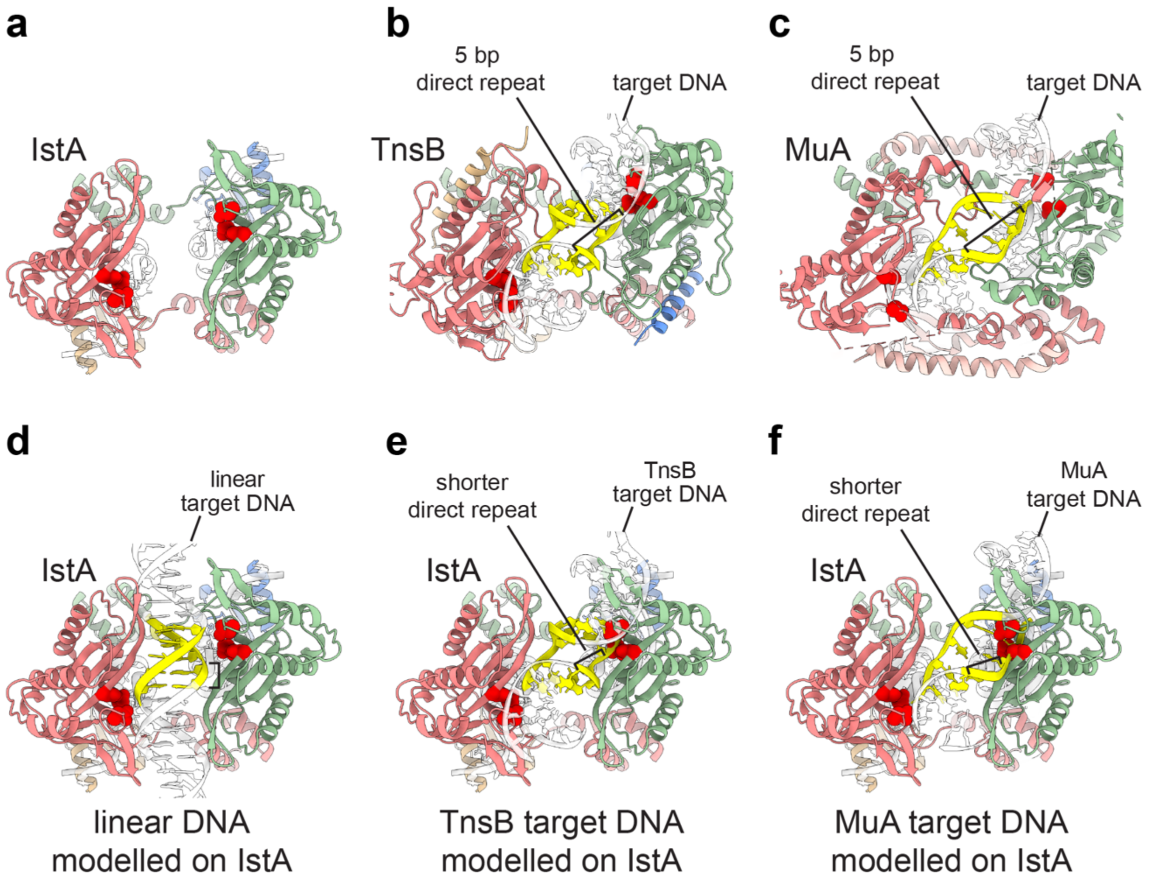

Supplementary Fig. 11 | Configuration of the active sites of IstA, TnsB and MuA. a, The active sites of the catalytic IstA monomers are 31 Å apart. DDE motifs are colored as red spheres though the figure. All structures of the figure have been aligned using the red IstA monomer as reference. **b,** The active sites of TnsB (PDB ID 8AA5 [https://doi.org/10.2210/pdb8AA5/pdb]), which are separated by 40 Å, generate staggered cuts separated by 5 bp (highlighted in yellow) in the target DNA (white). **c,** The catalytic DDE motifs of MuA (PDB ID 4FCY [https://doi.org/10.2210/pdb4FCY/pdb]) are separated by 44 Å and also produce staggered cuts that generate 5 bp repeats. **d,** Ideal linear target DNA modelled in the active site shows how the position of the IstA catalytic domains would generate a pair of cuts staggered ~2 bp apart (indicated with black segment) and, therefore, are offset from the positions needed to produce the characteristic 5 bp direct duplications of this element (for comparison 5 bases are colored in yellow). **e,** TnsB target DNA modelled on IstA shows that the configuration of the DDE motifs would generate shorter staggered cuts and, consequently, direct repeats (compare black segment with that shown in b). **f,** MuA target DNA modelled on IstA shows that the configuration of the DDE motifs would also generate shorter staggered cuts and direct repeats (compare black segment with that shown in c).

Supplementary Tables

Supplementary Table 1. Oligonucleotides used for the functional, biochemical and structural assays. Terminal repeats are underlined and catalytic adenosine colored red the first they appear in the table and for the cryo-EM substrates.

Name	Sequence (5' -> 3')

Integration assay: dependence on IstB and L *vs.* R

L-TIR-fwd	TGTTAAAGCCGATGATAAAATCCCCAATATAGCCGGAATAAAATTCCCCACTTAC
L-TIR-rev	GTAAGTGGGGAATTTTATTCCGGCTATATTGGGGATTTTATCATCGGCTTTAACA
R-TIR-fwd	CCTTCTGGGGAATTTTAAACCGGCGATTTTGGGGAAAAAATAATCGGCCTTGACA
R-TIR-rev	TGTCAAGGCCGATTATTTTTTCCCCAAAATCGCCGGTTTAAAATTCCCCAGAAGG

Fig. 1d.	TIR pre-cleaved duplexes were generated annealing L-TIR-fwd with L-TIR-rev, and R-TIR-fwd with R-TIR-rev. Both duplexes were mixed in equimolar concentrations (1 µM total).
Fig. 1e.	TIR pre-cleaved duplexes were generated annealing L-TIR-fwd with L-TIR-rev, and R-TIR-fwd with R-TIR-rev. Duplexes were used in isolation (L TIR, R TIR) or mixed in equimolar concentrations (L+R TIR).

Integration assay: donor length

L-TIR-fwd	TGTTAAAGCCGATGATAAAATCCCCAATATAGCCGGAATAAAATTCCCCACTTAC
L-TIR-rev	GTAAGTGGGGAATTTTATTCCGGCTATATTGGGGATTTTATCATCGGCTTTAACA
R-TIR-fwd	CCTTCTGGGGAATTTTAAACCGGCGATTTTGGGGAAAAAATAATCGGCCTTGACA
R-TIR-rev	TGTCAAGGCCGATTATTTTTTCCCCAAAATCGCCGGTTTAAAATTCCCCAGAAGG
L-TIR-38bp-fwd	TGTTAAAGCCGATGATAAAATCCCCAATATAGCCGGAA
L-TIR-38bp-rev	TTCCGGCTATATTGGGGATTTTATCATCGGCTTTAACA
L-TIR-27bp-fwd	TGTTAAAGCCGATGATAAAATCCCCAA
L-TIR-27bp-rev	TTGGGGATTTTATCATCGGCTTTAACA
L-TIR-15bp-fwd	TGTTAAAGCCGATGA
L-TIR-15bp-rev	TCATCGGCTTTAACA
R-TIR-38bp-fwd	AACCGGCGATTTTGGGGAAAAAATAATCGGCCTTGACA
R-TIR-38bp-rev	TGTCAAGGCCGATTATTTTTTCCCCAAAATCGCCGGTT
R-TIR-27bp-fwd	TTGGGGAAAAAATAATCGGCCTTGACA
R-TIR-27bp-rev	TGTCAAGGCCGATTATTTTTTCCCCAA
R-TIR-15bp-fwd	TAATCGGCCTTGACA
R-TIR-15bp-rev	TGTCAAGGCCGATTA

Fig. 1f.	TIR pre-cleaved duplexes of different length were obtained annealing the corresponding -fwd and -rev oligos (e.g. L-TIR-38bp-fwd with L-TIR-38bp-rev).<br>For each length, left and right duplexes were mixed in equimolar concentrations (1 µM total).

Integration assay: donor sequence dependence

L-TIR-fwd	TGTTAAAGCCGATGATAAAATCCCCAATATAGCCGGAATAAAATTCCCCACTTAC
L-TIR-rev	GTAAGTGGGGAATTTTATTCCGGCTATATTGGGGATTTTATCATCGGCTTTAACA
L-TIR-Over5bp-fwd	TGAATTGTTAAAGCCGATGATAAAATCCCCAATATAGCCGGAATAAAATTCCCCACTTAC
R-TIR-fwd	CCTTCTGGGGAATTTTAAACCGGCGATTTTGGGGAAAAAATAATCGGCCTTGACA
R-TIR-rev	TGTCAAGGCCGATTATTTTTTCCCCAAAATCGCCGGTTTAAAATTCCCCAGAAGG

R-TIR-Over5bp-rev	ATTCATGTCAAGGCCGATTATTTTTTCCCCAAAATCGCCGGTTTAAAATTCCCCAGAAGG
L-unprocessed-fwd	CAACGTGAATTGTTAAAGCCGATGATAAAATCCCCAATATAGCCGGAATAAAATTCCCCACTTAC
L-unprocessed-rev	GTAAGTGGGGAATTTTATTCCGGCTATATTGGGGATTTTATCATCGGCTTTAACAATTCACGTTG
R-unprocessed-fwd	CCTTCTGGGGAATTTTAAACCGGCGATTTTGGGGAAAAAATAATCGGCCTTGACATGAATCAACG
R-unprocessed-rev	CGTTGATTCATGTCAAGGCCGATTATTTTTTCCCCAAAATCGCCGGTTTAAAATTCCCCAGAAGG
Random-60bp-fwd (Rd60)	TGCTTGCGATGATCCGACGTGTTAGCCACGCTGACTAGTTATGCCATGCCTCCCTTCAGG
Random-60bp-rev (Rd60)	CCTGAAGGGAGGCATGGCATAACTAGTCAGCGTGGCTAACACGTCGGATCATCGCAAGCA

Fig. 1g.	Substrates were obtained as follow:<br><br>Blunt: L-TIR-fwd was annealed with L-TIR-rev and R-TIR-fwd was annealed with R-TIR-rev. Both pre-cleaved TIRs were mixed in an equimolar ratio (1 μM total).<br><br>5'overhang: L-TIR-Over5bp-fwd was annealed with L-TIR-rev and R-TIR-fwd was annealed with R-TIR-Over5bp-rev. Both pre-cleaved TIRs were mixed in an equimolar ratio (1 μM total).<br><br>unprocessed: L-unprocessed-fwd was annealed with L-unprocessed-rev and R-unprocessed-fwd was annealed with R-unprocessed-rev. Both unprocessed ends were mixed in an equimolar ratio (1 μM total).<br><br>random (Rd60): Random-60bp-fwd was annealed with Random-60bp-rev (1 μM).

Integration assay: overhang length

L-TIR-fwd	TGTTAAAGCCGATGATAAAATCCCCAATATAGCCGGAATAAAATTCCCCACTTAC
L-TIR-rev	GTAAGTGGGGAATTTTATTCCGGCTATATTGGGGATTTTATCATCGGCTTTAACA
L-TIR-Over6bp-fwd	GTGAATTGTTAAAGCCGATGATAAAATCCCCAATATAGCCGGAATAAAATTCCCCACTTAC
L-TIR-Over5bp-fwd	TGAATTGTTAAAGCCGATGATAAAATCCCCAATATAGCCGGAATAAAATTCCCCACTTAC
L-TIR-Over4bp-fwd	GAATTGTTAAAGCCGATGATAAAATCCCCAATATAGCCGGAATAAAATTCCCCACTTAC
L-TIR-Over3bp-fwd	AATTGTTAAAGCCGATGATAAAATCCCCAATATAGCCGGAATAAAATTCCCCACTTAC
R-TIR-fwd	CCTTCTGGGGAATTTTAAACCGGCGATTTTGGGGAAAAAATAATCGGCCTTGACA
R-TIR-rev	TGTCAAGGCCGATTATTTTTTCCCCAAAATCGCCGGTTTAAAATTCCCCAGAAGG
R-TIR-Over6bp-rev	CATTCATGTCAAGGCCGATTATTTTTTCCCCAAAATCGCCGGTTTAAAATTCCCCAGAAGG
R-TIR-Over5bp-rev	ATTCATGTCAAGGCCGATTATTTTTTCCCCAAAATCGCCGGTTTAAAATTCCCCAGAAGG
R-TIR-Over4bp-rev	TTCATGTCAAGGCCGATTATTTTTTCCCCAAAATCGCCGGTTTAAAATTCCCCAGAAGG
R-TIR-Over3bp-rev	TCATGTCAAGGCCGATTATTTTTTCCCCAAAATCGCCGGTTTAAAATTCCCCAGAAGG

Supplementary Fig. 2a.	L-TIR-rev was annealed with L-TIR-fwd to generate a pre-cleaved substrate with blunt ends. L-TIR-rev was annealed with L-TIR-Over6bp-fwd, L-TIR-Over5bp-fwd, L-TIR-Over4bp-fwd or L-TIR-Over3bp-fwd to generate pre-cleaved duplexes with different overhang lengths.<br><br>R-TIR-fwd was annealed with R-TIR-rev to generate a pre-cleaved substrate with blunt ends. R-TIR-fwd was annealed with R-TIR-Over6bp-rev, R-TIR-Over5bp-rev, R-TIR-Over4bp-rev or R-TIR-Over3bp-rev to generate pre-cleaved duplexes with different overhang lengths.<br><br>Left and right end pre-cleaved duplexes with similar overhang configurations were mixed in an equimolar ratio (1 μM total).
Supplementary Fig. 2b.	L-TIR-rev was annealed with L-TIR-Over5bp-fwd to generate a pre-cleaved substrate with a 5-base long 5'overhang.<br><br>R-TIR-fwd was annealed with R-TIR-Over5bp-rev to generate a pre-cleaved substrate with with a 5-base long 5'overhang.<br><br>Left and right end pre-cleaved duplexes were mixed in an equimolar ratio (1 μM total).

DNA binding. Fluorescence anisotropy

Fluo-R-TIR-fwd (R1-R2)	/FAM/CCTTCTGGGGAATTTTAAACCGGCGATTTTGGGGAAAAAAATAATCGGCCTTGACA
R-TIR-rev (R1-R2)	TGTCAAGGCCGATTATTTTTTCCCCAAAATCGCCGGTTTAAAATTCCCCAGAAGG
Fluo-R-TIR-27bp-fwd (R1)	/FAM/TTGGGGAAAAAAATAATCGGCCTTGACA
R-TIR-27bp-rev (R1)	TGTCAAGGCCGATTATTTTTTCCCCAA
Fluo-Random-60bp-fwd (Rd60)	/FAM/TGCTTGCGATGATCCGACGTGTTAGCCACGCTGACTAGTTATGCCATGCCTCCCTTCAGG
Random-60bp-rev (Rd60)	CCTGAAGGGAGGCATGGCATAACTAGTCAGCGTGGCTAACACGTCGGATCATCGCAAGCA
Fluo-Random-27bp-fwd (Rd27)	/FAM/TGCTAACACGTCGGATCATCGCAAGCA
Random-27bp-rev (Rd27)	TGCTTGCGATGATCCGACGTGTTAGCA

Fig. 2a.	R1-R2: Pre-cleaved substrate containing the full right TIR was generated annealing Fluo-R-TIR-fwd and R-TIR-rev.<br><br>R1: Pre-cleaved substrate containing the first right repeat was generated annealing Fluo-R-TIR-27bp-fwd and R-TIR-27bp-rev<br><br>Rd60: Random 60mer was obtained annealing Fluo-Random-60bp-fwd with Random-60bp-rev.<br><br>Rd27: Radom 27mer was obtained annealing Fluo-Random-27bp-fwd with Random-27bp-rev.

Analytical gel filtration chromatography

L-TIR-fwd	TGTTAAAGCCGATGATAAAATCCCCAATATAGCCGGAATAAAATTCCCCACTTAC
L-TIR-rev	GTAAGTGGGGAATTTTATTCCGGCTATATTGGGGATTTTATCATCGGCTTTAACA
R-TIR-fwd	CCTTCTGGGGAATTTTAAACCGGCGATTTTGGGGAAAAAATAATCGGCCTTGACA
R-TIR-rev	TGTCAAGGCCGATTATTTTTTCCCCAAAATCGCCGGTTTAAAATTCCCCAGAAGG
Biot-L-TIR-rev	/biot/GTAAGTGGGGAATTTTATTCCGGCTATATTGGGGATTTTATCATCGGCTTTAACA
Biot-R-TIR-fwd	/biot/CCTTCTGGGGAATTTTAAACCGGCGATTTTGGGGAAAAAATAATCGGCCTTGACA

Fig. 2b.	TIR pre-cleaved duplexes were generated annealing L-TIR-fwd with L-TIR-rev, and R-TIR-fwd with R-TIR-rev. Both duplexes were mixed in equimolar concentrations (1 µM total).<br><br>Biotinilated TIR pre-cleaved duplexes were generated annealing L-TIR-fwd with Biot-L-TIR-rev, and Biot-R-TIR-fwd with R-TIR-rev. Both duplexes were mixed in equimolar concentrations (1 µM total).

Preliminary cryo-electron microscopy studies

L-TIR-Over5bp-fwd	TGAATTGTTAAAGCCGATGATAAAATCCCCAATATAGCCGGAATAAAATTCCCCACTTAC
L-TIR-rev	GTAAGTGGGGAATTTTATTCCGGCTATATTGGGGATTTTATCATCGGCTTTAACA
R-TIR-fwd	CCTTCTGGGGAATTTTAAACCGGCGATTTTGGGGAAAAAATAATCGGCCTTGACA
R-TIR-Over5bp-rev	ATTCATGTCAAGGCCGATTATTTTTTCCCCAAAATCGCCGGTTTAAAATTCCCCAGAAGG

Supplementary Fig. 4.	Pre-cleaved right TIR duplex for structural studies was generated annealing R-TIR-fwd with R-TIR-Over5bp-rev.

High-resolution cryo-electron microscopy

R-TIR-fwd	CCTTCTGGGGAATTTTAAACCGGCGATTTTGGGGAAAAAATAATCGGCCTTGACA
R-TIR-Over5bp-rev	ATTCATGTCAAGGCCGATTATTTTTTCCCCAAAATCGCCGGTTTAAAATTCCCCAGAAGG

Fig. 3.	Pre-cleaved right TIR duplex for structural studies was generated annealing R-TIR-fwd with R-TIR-Over5bp-rev.

Supplementary Table 2. Cryo-EM data collection, refinement and validation statistics

	IstA cleaved donor complex<br>(EMD-15848)<br>(PDB 8B4H)
**Data collection and processing**	
Microscope	Titan Krios (DLS)
Detector	BioQuantum-K3
Voltage (kV)	300
Electron dose (e/A^2)	59.7
Dose rate (e/A^2/fraction)	1.19
Nominal Defocus range (µm)	-1.2 to -2.7
Nominal magnification	81,000 x
Pixel size (Å)	1.06
Pixel size super-resolution (Å)	0.53
Total of exposure time (s)	3.36
Number of micrographs	7,215
Total extracted particles (no.)	11,100,000
Final particle images (no.)	337,376
Symmetry imposed	C2
Resolution (per 0.143 FSC) (Å)	3.4
Applied B-factor (Å^2)	-115.8
**Refinement and validation**	
Non-hydrogens / Hydrogens atoms	31,138 / 14,570
Protein residues / Nucleotides	1,440 / 224
Ligands (Mg^{2+})	2
All-atom clashscore	11.08
MolProbity score	1.7
Cb outliers (%)	0.0
RMS deviations	
Bond lengths (Å)	0.004
Bond angles (°)	0.706
Ramachandran (%)	
Outliers	0.00
Allowed	2.73
Favored	97.27
Rotamer outliers (%)	0.0
